# A web-based application for diabetes subtyping: The DDZ Diabetes-Cluster-Tool

**DOI:** 10.1007/s00592-024-02436-5

**Published:** 2025-01-17

**Authors:** Tim Mori, Katsiaryna Prystupa, Klaus Straßburger, Marc Bonn, Oana Patricia Zaharia, Olaf Spörkel, Oliver Kuß, Michael Roden, Robert Wagner

**Affiliations:** 1 Institute for Biometrics and Epidemiology, German Diabetes Center, Leibniz Center for Diabetes Research at Heinrich Heine University Düsseldorf, Auf’m Hennekamp 65, 40225 Düsseldorf, Germany; 2 German Center for Diabetes Research (DZD), Partner Düsseldorf, München-Neuherberg, Germany; 3 Institute for Clinical Diabetology, German Diabetes Center, Leibniz Center for Diabetes Research at Heinrich Heine University, Düsseldorf, Germany; 4 National Diabetes Information Center, German Diabetes Center, Leibniz Center for Diabetes Research at Heinrich Heine University, Düsseldorf, Germany; 5 Division of Endocrinology and Diabetology, Medical Faculty, University Hospital Düsseldorf, Heinrich Heine University Düsseldorf, Düsseldorf, Germany; 6 Centre for Health and Society, Faculty of Medicine, Heinrich Heine University Düsseldorf, Düsseldorf, Germany

Adult-onset diabetes mellitus is a heterogeneous disease with substantial variability across clinical phenotypes [1]. A phenotype-based clustering approach classifies people with diabetes into more granular subtypes [1] and has been replicated in different populations [2]. These novel subtypes comprise severe autoimmune diabetes (SAID), severe insulin-deficient diabetes (SIDD), severe insulin-resistant diabetes (SIRD), mild obesity-related diabetes (MOD) and mild age-related diabetes (MARD) [1]. They not only differ in their clinical characteristics such as age at diagnosis, HbA1c, body mass index (BMI), β-cell function (from homeostasis model assessment: HOMA2-B) and insulin resistance (from HOMA2-IR), but also in terms of diabetes-related complications [1, 2]. For example, compared to individuals in the MARD cluster, individuals in the SIDD cluster had a higher hazard rate for retinopathy and individuals in the SIRD cluster had a higher hazard rate for chronic kidney disease and steatotic liver disease [1]. While precision diabetes diagnosis holds a promise for precision treatment of adult-onset diabetes, evidence is still needed to show a benefit of cluster-targeted therapy compared to treatments based on current guidelines. One additional obstacle to adopt this method into clinical practice is a relatively complex cluster assignment algorithm (also referred to as “nearest centroid approach” [2]), which is necessary to identify a person’s diabetes subtype. The goal of the project described here was to develop an easy-to-use online tool for the classification of individuals with new-onset diabetes into the clusters [1]. A second goal was to provide a simple graphical measure of cluster similarity, which can help clinicians assess the closeness of a given individual to the assigned subtype.

To this end, the German Diabetes Center (DDZ) developed the DDZ Diabetes-Cluster-Tool, which was launched in May 2023. This tool is available at https://diabetescalculator.ddz.de/diabetescluster-en/ in English and German language. It was developed with the statistical software R version 4.2.0 [3] and the Shiny web framework. In the menu, the user can directly input all clinical and laboratory data necessary for cluster assignment: presence of antibodies to glutamic acid decarboxylase (GAD), age at diagnosis, BMI, fasting plasma glucose, fasting C-peptide, HbA1c and sex. Note that fasting plasma glucose and C-peptide values are used to compute the HOMA2-B and HOMA2-IR values, which are needed for cluster assignment. For ease of use, the tool supports multiple unit formats for inputting clinical data. For example, fasting plasma glucose can be supplied as either mmol/l or mg/dl. After entering the information, the cluster assignment is computed automatically with the nearest centroid approach using the centroid and normalization information from the All-New Diabetes in Scania (ANDIS) cohort [1]. In the output, the user receives two pieces of information, the diabetes subtype and the “degree of similarity” to each of the five subtypes presented as bar chart.

**Fig. 1:**
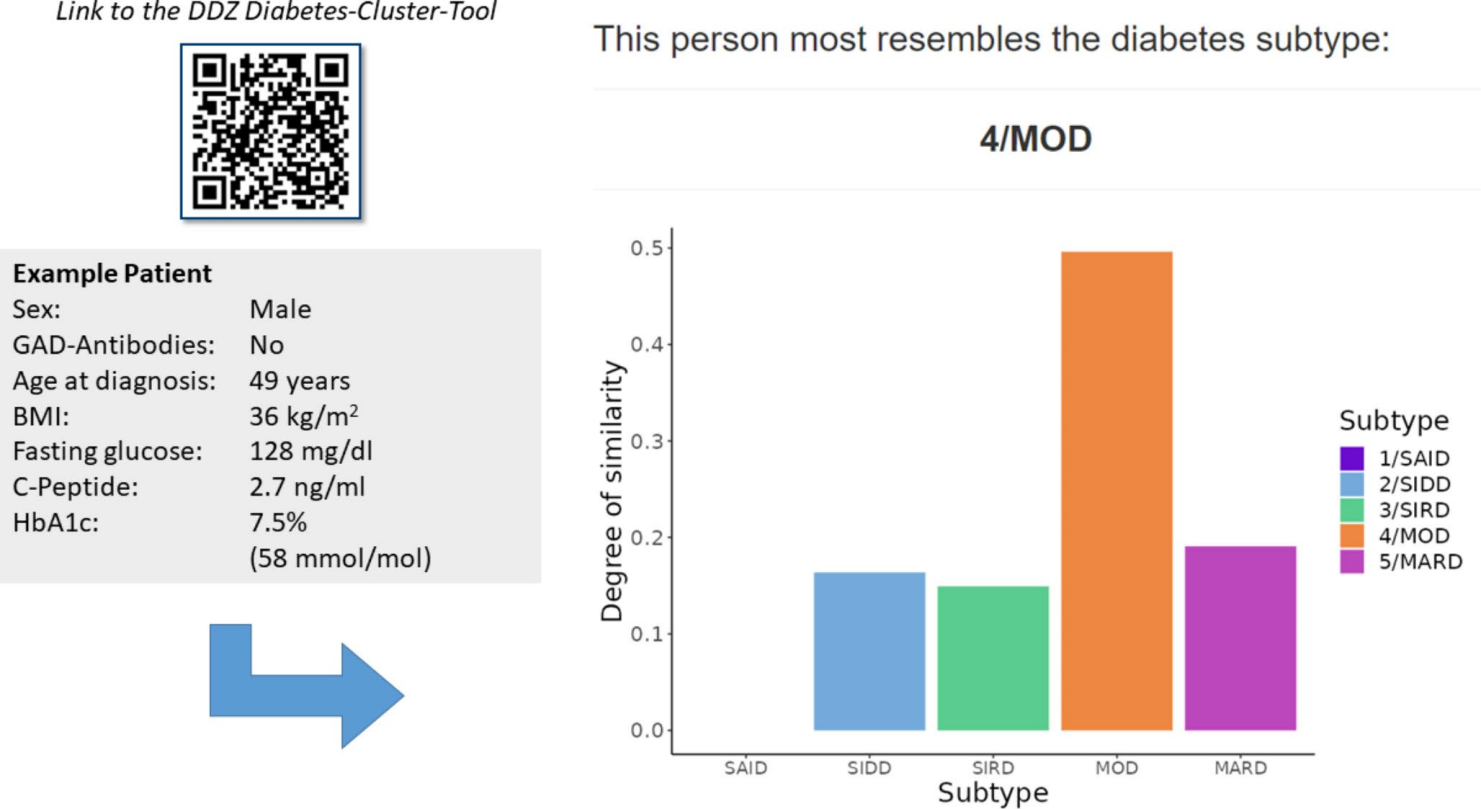
A fictional example of an individual and their output of the DDZ Diabetes-Cluster-Tool, including the assigned subtype and the degree of similarity to each of the five subtypes. The QR code contains the link to the web application, which can be used on both mobile devices and desktop computers

The degree of similarity is computed based on the distance of the individual to each of the five centroids taking into account all of the entered clinical information. For ease of interpretation, the distances are inverted so that the highest bar corresponds to the shortest distance and thus the best fit. Moreover, the distances are scaled so that they add up to 1, which makes it easier to compare the subtype similarity between individuals. This simple visualization allows the user to identify “borderline cases” who are similar to several clusters, i.e. where there is not one clear subtype to describe the observed phenotype. Note that the degree of similarity to the SAID cluster will always be either 0 or 1, because assignment to this cluster is determined categorically by the absence or presence of GAD-antibodies.

Figure 1 shows a fictional example of an individual and their output of the DDZ Diabetes-Cluster-Tool. The assigned subtype is MOD and the individual’s phenotype seems to match this cluster well, as indicated by the high degree of similarity. Note that this example individual is rather young but highly obese, as is typical for the MOD subtype [1]. The degree of similarity with the other clusters is considerably smaller, which reflects a larger distance to the respective centroids (e.g. MARD). Although current guidelines do not yet advocate the diabetes clusters, the tool could provide information about the pathophysiology for clinicians to discuss the diabetes phenotype of an individual presenting in clinical practice. Moreover, researchers can use this tool to classify individuals and further advance our insights into the underlying differences between the subtypes.

While diabetes subtypes provide an intuitive framework for understanding disease heterogeneity, it is important to keep in mind their limitations. Subclassification into discrete clusters always results in a loss of information compared to when the corresponding individual clinical features are considered on a continuous scale. While simplification aids interpretation and communication, specific prediction models should utilize the continuous, individual clinical features [4]. An alternative approach proposed by other researchers is to display the heterogeneity of diabetes in a continuous manner using a tree-like graph structure [5]. A further limitation of the clusters is that they were developed using data at diabetes diagnosis and clustering based on data obtained at subsequent time-points of disease course could lead to a change of cluster assignment [2]. Finally, individuals within a cluster may still be relatively heterogeneous and some individuals may not clearly belong to any of the clusters. For such individuals, who may present clinical features overlapping with several subtypes, the discrete cluster assignment will likely be less informative. The DDZ Diabetes-Cluster-Tool includes a graphical measure of cluster similarity to help identify such cases.

Overall, the phenotype-based adult-onset diabetes clusters have received considerable attention. With the DDZ Diabetes-Cluster-Tool we provide an easy-to-use web-based application for the technical implementation of diabetes cluster assignments.

## Data Availability

The R code for the DDZ Diabetes-Cluster-Tool is available upon reasonable request.
